# Exploring the Prospective Role of Propolis in Modifying Aging Hallmarks

**DOI:** 10.3390/cells13050390

**Published:** 2024-02-24

**Authors:** Carla Scorza, Valeria Goncalves, Josef Finsterer, Fúlvio Scorza, Fernando Fonseca

**Affiliations:** 1 Disciplina de Neurociência, Departamento de Neurologia e Neurocirurgia, Universidade Federal de São Paulo (UNIFESP), São Paulo 04039-032, Brazil; vaal.cassia@gmail.com (V.G.); scorza@unifesp.br (F.S.); 2 Neurology and Neurophysiology Center, 1180 Vienna, Austria; fifigs1@yahoo.de; 3 Laboratório de Análises Clínicas da Faculdade de Medicina do ABC, Santo André 09060-650, Brazil; profferfonseca@gmail.com; 4 Departamento de Ciencias Farmaceuticas, Universidade Federal de Sao Paulo (UNIFESP), Diadema 09972-270, Brazil

**Keywords:** propolis, aging, age-related diseases

## Abstract

Aging populations worldwide are placing age-related diseases at the forefront of the research agenda. The therapeutic potential of natural substances, especially propolis and its components, has led to these products being promising agents for alleviating several cellular and molecular-level changes associated with age-related diseases. With this in mind, scientists have introduced a contextual framework to guide future aging research, called the hallmarks of aging. This framework encompasses various mechanisms including genomic instability, epigenetic changes, mitochondrial dysfunction, inflammation, impaired nutrient sensing, and altered intercellular communication. Propolis, with its rich array of bioactive compounds, functions as a potent functional food, modulating metabolism, gut microbiota, inflammation, and immune response, offering significant health benefits. Studies emphasize propolis’ properties, such as antitumor, cardioprotective, and neuroprotective effects, as well as its ability to mitigate inflammation, oxidative stress, DNA damage, and pathogenic gut bacteria growth. This article underscores current scientific evidence supporting propolis’ role in controlling molecular and cellular characteristics linked to aging and its hallmarks, hypothesizing its potential in geroscience research. The aim is to discover novel therapeutic strategies to improve health and quality of life in older individuals, addressing existing deficits and perspectives in this research area.

## 1. Introduction

It is estimated that the global population of people over the age of 64 years will more than double, increasing from 761 million in 2021 to 1.6 billion by 2050 [1]. The segment comprising individuals who are 80 years and beyond is experiencing a more accelerated rate of expansion. When it comes to acknowledging the significance of aging societies, apprehension tends to be the prevailing reaction. Expressions such as “grey tsunami” or “silver tsunami” insinuate that a larger population of older individuals will impose a burden on societies, especially considering the limited success of global endeavors in extending the period of healthy life [2]. At present, medical interventions primarily increase lifespan by prolonging the duration of morbidity [3]. An interesting aspect is that, despite the upward trend in average human life expectancy, there has been no corresponding rise in the maximum lifespan. Moreover, there is a considerable diversity in aging rates within a given species. Senescence is genetically rooted, but it is possible to modulate a species’ genetically determined lifespan by manipulating genes or adjusting dietary habits [4,5,6]. Taking queen honey bees as a case in point, their lifespan is typically around ten times more extensive than that of worker bees, despite possessing identical genetic material [7]. In this setting, multiple studies consistently provide undeniable proof of the foundational relevance of gene–diet interactions in molding individual disparities in health outcomes and longevity [7,8,9,10,11].

Aging is a broad concept that refers to the gradual decline in functionality that is commonly observed in most living organisms as they grow older [12,13]. It is outlined as a weakened response to stress, an amplification of homeostatic instability, and a heightened susceptibility to age-related diseases. Aging coincides with conditions like type 2 diabetes, cancer, and cardiovascular and neurodegenerative diseases, among many others [14].

Significant progress in research has provided critical knowledge about the processes and intrinsic molecular and cellular factors behind the biology of aging [15,16,17,18,19,20,21]. Referred to as hallmarks of aging, many factors implicated in the aging process have been identified, involving persistent inflammation, mitochondrial dysfunction, genomic instability, telomere shortening, epigenetic modifications, impaired protein homeostasis, compromised autophagy, dysregulated nutrient sensing, disrupted intercellular communication, and imbalance of gut microbiota, among other emerging factors [22]. Although aging and age-related diseases present clinically distinct characteristics, research findings uphold support for the concept that both arise as a result of distinctive combinations of changes within a limited set of fundamental biological processes [23]. While the hallmarks of aging manifest in all individuals, the precise interplay among these mechanisms and their collective contribution or causal relationships in the broader aging process and the emergence of age-related diseases are not fully understood. Nevertheless, these hallmarks may present a more holistic insight into both aging itself and age-related diseases, paving the way for research in interventions and therapies to enhance health in older individuals [24]. Incorporating environmental interventions, particularly nutrition, for the purpose of regulating the fundamental biological pillars of aging and age-related diseases has ignited scientific attention and propelled research efforts in the biomedical sphere. Propolis, together with its bioactive constituents, represents an emerging bioresource with prospects in the nutraceutical and pharmaceutical fields [25,26,27,28,29,30]. In light of this, propolis is acknowledged for its potential to serve as a foundation for the formulation of innovative pharmaceuticals.

**Figure 1:**
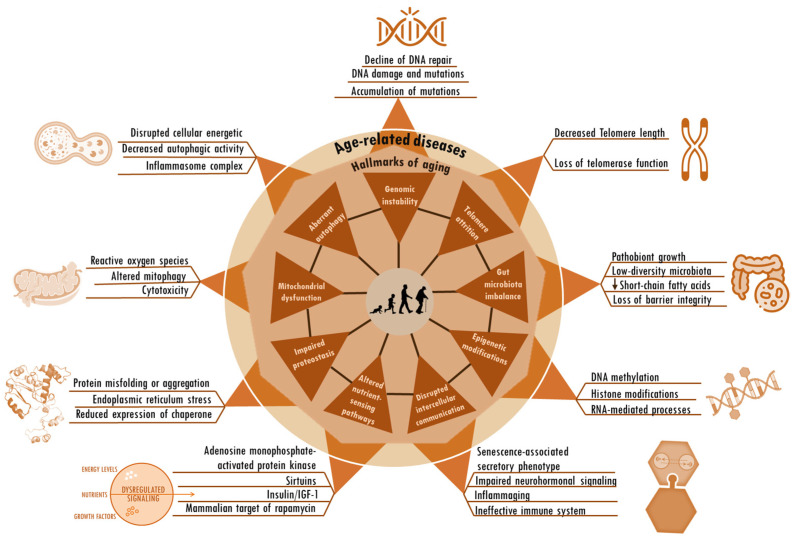
Hallmarks of aging and age-related alterations. The hallmarks of aging, including genomic instability, telomere attrition, aberrant autophagy, gut microbiota imbalance, mitochondrial dysfunction, epigenetic modifications, altered nutrient-sensing pathways, impaired protein homeostasis, and disrupted intercellular communication, are deeply interconnected. These same factors seem to be root cause contributors to age-related diseases. The particular factors and sequences that trigger the shift from the normal changes of aging to the onset of age-related diseases remain unknown.

This article is centered on recent scientific evidence that endorses propolis and its bioactive components’ capacity to regulate the primary cellular and molecular traits linked to aging (Figure 1). These characteristics of aging are also relevant to age-related diseases. We posit that the existing pool of evidence positions these bee-derived substances as promising contenders in the field of geroscience research, with the goal of discovering novel therapeutic strategies to improve health and quality of life in old age. We also tackle the shortcomings and prospects in this area of investigation.

## 2. Propolis

**Figure 2:**
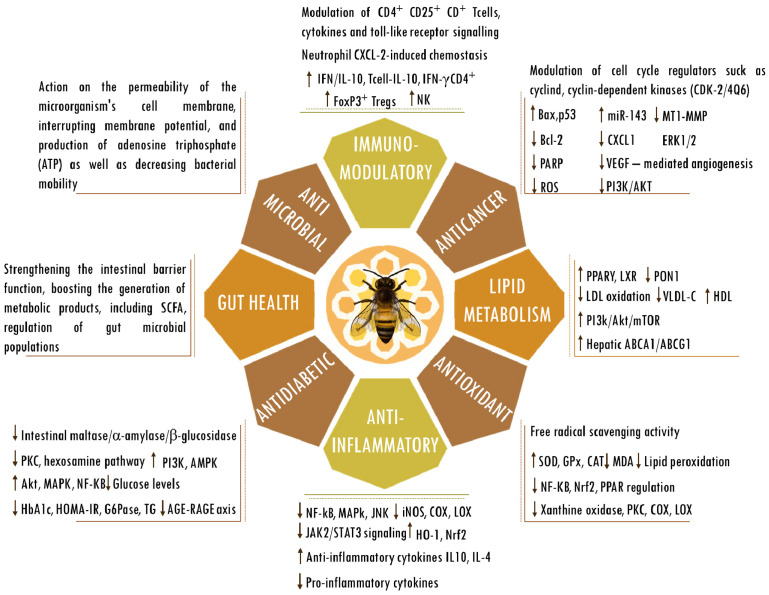
Biological activities of propolis. Abbreviations: ABCA1, ATP-binding cassette subfamily A member 1; ABCG1, ATP-binding cassette (ABC) subfamily G member 1; AGEs, advanced glycation end products; Bax, bcl-2-like protein 4; Bcl-2, B cell lymphoma 2; CAT, catalase; CD, cluster of differentiation; COX; cyclooxygenase; CXCL-2, chemokine (C-X-C motif) ligand 2; ERK, extracellular signal-regulated kinase; FOXP3, forkhead box P3; G6Pase, glucose-6-phosphatase; GPx, glutathione peroxidase; HbA1C, hemoglobin A1C; HDL, high-density lipoprotein; HO-1, heme oxygenase-1; HOMA-IR, homeostasis model assessment-estimated insulin resistance; iNOS, inducible nitrogen oxide synthase; IFN, interferon; IL, interleukin; JAK2/STAT3, Janus kinase 2/signal transducer and activator of transcription 3; JNK, Jun kinase; LDL, low-density lipoprotein; LOX; lipoxygenase; LXRs, liver X receptors; MDA, malondialdehyde; mTOR, mammalian target of rapamycin; MT1-MMP, membrane type 1-matrix metalloproteinase; miR143, microRNA 143; NF-κB, nuclear factor kappa-light-chain-enhancer of activated B cells; NK, natural killer cells; Nrf2, nuclear factor erythroid 2-related factor 2; PPARγ, peroxisome proliferator-activated receptor gamma; PARP, poly (ADP-ribose) polymerase; p53, tumor protein p53; PI3K/AKT, phosphatidylinositol 3-kinase/ protein kinase B; PKC, protein kinase C; PON1, paraoxonase-1; RAGE, receptor for AGEs; regulatory T cells (Tregs); ROS, reactive oxygen species; SCFAs, short-chain fatty acids; SOD, superoxide dismutase; TG, triglyceride; VEGF, vascular endothelial growth factor; VLDL-c, very-low-density lipoprotein cholesterol. The up and down arrows indicate an increase or reduction in the mentioned components.

Bees produce propolis, a resinous substance composed of plant elements, and apply it in stratified layers within hives to seal cracks, protect against microorganisms, regulate temperature, and encapsulate deceased intruders to prevent contamination of the colonies [31,32]. A propolis-enriched environment in bee colonies has been observed to impact bacterial populations and contribute to the stability of the bee microbiota [33]. Produced by several cataloged species, the composition of propolis varies according to geography, bee species, and local flora. Despite the potential variation among its more than 800 compounds, the samples show similarities [34]. Moreover, propolis is rich in vital vitamins, including A, D, and those from the B complex, as well as essential minerals such as calcium, magnesium, potassium, manganese, zinc, and iron. Additionally, it comprises specific fatty acids, enzymes originating from bee saliva, polysaccharides, and sugars that are frequently identified in propolis [35,36]. In the realm of organic constituents, noteworthy elements include terpenes and steroids, as well as sugars and amino acids, along with various flavonoids. Notable among the flavonoids are caffeic, cinnamic, p-coumaric, and chicoric acids, as well as quercetin, pinocembrin, baicalin, galangin, and chrysin, owing to their significant antiglycation [37,38,39], cardioprotective [40,41,42,43,44], neuroprotective [45,46,47,48,49,50], cytostatic [51,52,53], radioprotective [54,55,56,57], antimicrobial [58,59,60,61,62,63], anticancer [64,65,66,67,68,69,70], antioxidant [71,72,73,74,75,76], immunomodulatory [77,78,79,80,81,82,83], antidiabetic [84,85,86,87,88,89], and anti-inflammatory [90,91,92,93,94,95] activities and influence on gut health [80,85,96,97,98] and lipid metabolism [99,100,101,102,103,104] (Figure 2). Nevertheless, the precise mechanisms through which propolis’s bioactive constituents operate remain incompletely understood. Studies tie their effects to the mitigation of mitochondrial dysfunction, oxidative stress, inflammation, and the scavenging of free radicals. Under normal physiological circumstances, reactive oxygen species serve as cellular signaling entities involved in processes such as cell growth, adhesion, differentiation, senescence, and apoptosis. However, heightened production of oxidants is indicative of progressing inflammation [105]. Ongoing oxidative stress and inflammation are common denominators in various pathologies, including cancer, cardiovascular diseases, diabetes, neurological disorders, psychiatric illnesses, kidney diseases, lung diseases, and aging [106,107]. Elevated levels of oxidative stress, quantifiable through biomarkers, have been observed in elderly individuals or those with unhealthy lifestyles, characterized by the consumption of unwholesome diets, tobacco use, alcohol consumption, lack of physical exercise, and genetic predisposition [106,107]. The concerted action of antioxidants in ameliorating the deleterious effects of oxidative stress is achieved through the activities of antioxidant enzymes and vitamins C and E, as well as flavonoids, such as those found in propolis [108,109]. Numerous studies substantiate the potential roles and effectiveness of propolis and its compounds in counteracting the detrimental effects associated with various acute and chronic diseases [25,26,27,28,110,111,112]. The therapeutic abilities of propolis and its active components in pharmacological applications and medicine have been affirmed. Ongoing research is also rooted in a more in-depth understanding of its biological activities.

## 3. Genomic Stability

A stable genome is crucial for cells to reliably pass on genetic information. Accumulating genome damage and somatic mutations that induce genome instability stand out as the primary mechanisms driving aging [113]. The aging process is associated with an observed escalation in the frequency of damages to DNA (i.e., single- and double-strand breaks, crosslinks, modified bases, oxidative-induced, and depurination or depyrimidination of sugars) and mutations (i.e., insertions, deletions, or substitutions), which contribute to age-related genomic instability [114]. Cell cycle stress, alterations in gene expression, and modifications in gene regulation are outcomes of genomic instability. A broad spectrum of alterations occurs at the molecular level, including the overexpression of proinflammatory genes and inflammatory adipocytokines, impaired metabolism of glycans, glucose, and fatty acids, increases in collagen crosslinking, abnormal insulin-IGF1, mitochondrial dysfunction, disturbance of germline genetic heterogeneity, dysbiosis, and dysregulation of signaling pathways such as FoxO, heat shock proteins, NF-Κb, and mTOR signaling [14,115]. Actually, genomic instability is functionally intertwined with every hallmark of aging [116]. In the end, this might account for cellular degeneration and functional decline that occur with age. The ultimate results of genomic instability include aging and the manifestation of age-related diseases.

Genomic instability is pivotal in both the initiation and progression of cancer [117]. For instance, a visible manifestation of aging-related DNA damage is sun-damaged skin. The formation of ultraviolet light (UV)-induced DNA mutations is a crucial contributing factor to the elevated risk of cancer development associated with age [118]. Studies have shown that apigenin, an active component present in propolis, plays a crucial role in safeguarding skin keratinocytes from ultraviolet B-induced DNA damage and oxidative stress [119]. This protective effect is achieved through the activation of nucleotide excision repair genes, facilitation of cyclobutane ring repair, inhibition of ROS production, and downregulation of NF-κB and MAPK signaling pathways. The protective properties of propolis extracts are evident in human experimental in vitro skin models, where they effectively inhibit UV-induced photodamage by mitigating the occurrence of DNA strand breaks and enhancing cell viability [120]. For instance, propolis is acknowledged as a powerful inhibitor of tyrosinase, a pivotal enzyme in the melanogenesis pathway [121]. Given that UV irradiation can trigger oxidative stress leading to DNA damage and pigmentation disorders, Stavropoulou et al. examined various samples of Greek propolis. Their evaluation involved assessing free radical scavenging, anticollagenase, and antityrosinase activities to determine the antioxidant and anti-aging attributes of propolis. The study results underscored that samples featuring higher levels of flavonoids showcased superior antioxidant activity and noteworthy collagenase inhibition. In contrast, samples rich in terpenoids showed heightened antityrosinase activity [122]. Compelling evidence supports the roles of propolis and its polyphenolic components in combating cancer by countering events such as elevated DNA damage, mutations, strand breaks, chromosomal instability, deregulated DNA repair machinery, impaired cell cycle checkpoints, and dysregulated homologous recombination [123]. Recent research indicates a notable escalation in age-related hepatocellular carcinoma among older individuals [124]. Cigarette smoke-derived carcinogens such as 4-aminobiphenyl (ABP) contribute to the development of hepatocellular carcinoma in humans. The enzyme cytochrome P450 family 2 subfamily E member 1 (CYP2E1), present across mammalian species, can trigger procarcinogens and convert certain specific drugs into cytotoxic products [125]. According to research findings, propolis treatment can inhibit the conversion of the tobacco carcinogen 4-aminobiphenyl (4-ABP) into its metabolite, N-hydroxy-ABP, by inhibiting CYP2E1 expression [126]. This results in reduced production of reactive oxygen species (ROS) and genotoxicity, serving as a protective measure against cancer development. The induction of mutation, cell death, malformation, and cancer is a well-known consequence of ionizing radiation on both somatic and germ cells. Flavonoids are important components of propolis that have neuroprotective effects against brain damage caused by ionizing radiation, attributed in part to their ability to scavenge free radicals and stabilize the DNA double-helix structure [127]. A primary mechanism widely documented to counteract toxic molecules is the removal of reactive oxygen species (ROS) by antioxidants, preventing their interaction with other molecules, including DNA [118]. Studies have confirmed the powerful antioxidant capacity of propolis and its components. The SH-SY5Y cell line is frequently employed as an in vitro experimental model for investigating Parkinson’s disease. A study reported that pretreatment of SH-SY5Y cells with Brazilian green propolis decreases H_2_O_2_-generated ROS derived from mitochondria along with a reduction in the signal intensity of 8-oxo-2′-deoxyguanosine (8-oxo-dG lesion is highly mutagenic) [128].

The issue of fertility preservation has gained significant attention in the context of cancer treatment. In a mouse study, in addition to restoring testicular testosterone levels, Indian propolis also alleviated testicular toxicity caused by mitomycin-C by decreasing DNA damage and enhancing antioxidant activity [129]. In alignment with this, a study examining the genotoxicity, cytotoxicity, and clonogenic death of Chinese hamster ovary cells exposed to gamma radiation indicated the potential utility of propolis in mitigating the adverse effects caused by ionizing radiation [130]. It has been observed that apigenin becomes integrated into the nuclear matrix of prostate epithelial cells. Its binding to nucleic acid bases within the matrix is believed to underlie its antioxidant and chemopreventive activities [131].

Iron’s impact on the aging process is frequently underestimated. Despite its importance for living organisms, its reactivity poses a potential risk [132]. With aging, there is a buildup of iron that correlates with the onset of many age-related diseases. Through its strong iron-binding ability and high lipophilicity, caffeic acid phenethyl ester (CAPE), a constituent of propolis, offers protection against cellular DNA damage caused by iron [133]. Studies have demonstrated a correlation between the antioxidant effects of Portuguese propolis extracts and their ability to prevent DNA damage induced by Fe^2+^ [134].

Diabetes emerges as a key health issue affecting the elderly population. The literature findings suggest that the use of propolis and epigallocatechin gallate results in a significant increase in the survival rate of diabetic mice [135]. Moreover, the administration of this dietary supplementation yields a notable reduction in lipid peroxidation levels in the kidney, liver, and brain tissue, along with reduced DNA damage in the peripheral lymphocytes of diabetic animals [135].

## 4. Telomere Length

Unique genomic segments called telomeres reside at the extremities of linear chromosomes [136]. Their primary function is to ensure chromosome integrity and genome stability, offering protection against harm and deterioration [137]. Without a telomere maintenance mechanism, telomeres in adult somatic cells undergo a reduction in length after each round of mitotic cell division, ultimately resulting in cellular senescence and aging [138]. The length of telomeres, a complex hereditary characteristic, imposes a significant constraint on telomere function and is intricately tied to the aging process and age-related diseases [139]. Lifestyle stress and environmental factors, such as diet, can influence telomere length [140]. The results of research involving human subjects showed that prolonged and frequent intake of bee products, including propolis, is linked to telomere length [141]. In Nasir et al.’s (2015) study, which involved comparing beekeepers consuming bee products with non-beekeeper control subjects abstaining from bee products and beekeeping-related activities, it was observed that annual bee product consumption correlated with an average increase of 0.258 kbp in telomere length [141]. The results also revealed that a greater daily frequency of bee product intake was associated with an average increment of 2.66 kbp in telomere length [141]. It has been suggested that telomere length might benefit positively from an antioxidant-rich diet, as telomeres are known to be prone to shortening due to ROS-induced damage, as well as being particularly vulnerable to the prevalent lesion 8-oxo-guanine and its accumulation [142].

Quercetin is a major flavonoid compound found in propolis that has gained significant recognition for its antioxidant and anti-inflammatory properties, along with its potential anticancer and antitumor effects [143]. For instance, targeting human telomeric G-quadruplex DNA represents one of the mechanisms through which this propolis component demonstrates its anticancer activity [144]. These findings propose quercetin as a strong contender for telomere targeting and as a potent anticancer agent. Additionally, quercetin acts as a suppressor of telomerase, an enzyme responsible for the maintenance of telomere integrity and length [145,146,147]. Telomerase overexpression, a characteristic feature in almost all human cancers, plays a key role in sustaining telomere length and contributes to abnormal cell proliferation and immortalization [148]. Caffeic acid phenethyl ester (CAPE), another potent bioactive compound found in propolis, also exhibits antitumoral and antioxidant properties, as well as cytotoxic and apoptotic effects. The induction of apoptosis by CAPE has been linked to its ability to induce the activity of the catalytic subunit of telomerase, known as human telomerase reverse transcriptase [149]. In parallel, by specifically targeting leukemia cells derived from leukemia patients, Manisa propolis effectively reduces the expression of human telomerase reverse transcriptase, with chrysin identified as a key constituent responsible for this effect [150,151]. Telomerase activation in cancer has been attributed to several mechanisms, including the involvement of oncogenes like Wnt, which serve as transcriptional regulators of telomerase [148]. The ability of Aydın Turkish propolis to modulate the expression of microRNAs in glioblastoma and brain cancer stem cells underscores its anticancer effect, with the WNT and NOTCH pathways being implicated [152].

## 5. Gut Microbiota

The microbiota is the distinctive combination of living microorganisms existing in a particular environment, for instance, the gut microbiota, while the microbiome encompasses all the microbial genomes within that habitat [153]. In addition to providing a nutrient-rich environment to the microbiota, the ecosystem resulting from the intricate symbiotic relationship between the host and the gut microbiota influences several key functions within the mammalian host, encompassing homeostasis, nutritional responses, metabolism, and immunity [154]. The mounting evidence indicates that disruptions in host–microbe symbiosis can cause compromised gut permeability, allowing gut microbiome-derived metabolites to reach the systemic circulation, triggering an inflammatory state, oxidative stress, and systemic immune responses [155]. Microbiota disruptions can negatively impact body processes and significantly increase the risk of developing diseases such as cancer, neurodegenerative diseases, and heart disease [156]. Evidence points to the gut microbiota’s active involvement in the age-related regulation of host gut inflammation and barrier permeability [157].

A rise in microbiota diversity is observed during the transition from childhood to adulthood, followed by a decline in individuals aged over 65 [156,158]. Starting in middle/late adulthood, intestinal microbiomes undergo a transformation towards greater uniqueness as people age [159]. In contrast, this deviation does not occur in less healthy subjects, and the unique measure of low gut microbiota is an indicator of increased mortality risk in a 4-year follow-up period [159]. Generally, the microbiota of older individuals is marked by reduced diversity, a prevalence of phyla Bacteroidetes over Firmicutes, an upsurge in opportunistic enteropathogens, and a decline in short-chain fatty acid (SCFA)-producing bacteria [160]. Cohort investigations focusing on the elderly reveal a greater prevalence of core genera, namely Bacteroides, Parabacteroides, and Alistipes, in individuals aged 65 years and older in comparison to younger, healthy counterparts [161]. Other research findings indicate a reduction in anaerobic species like Bifidobacteria, coupled with a rise in Lactobacillaceae and Enterobacteriaceae, including Escherichia coli, among the elderly [162]. Alongside a reduction in microbial diversity, shifts in the composition of gut commensals involve a prominent increase in opportunistic and potentially proinflammatory commensal microbes, accompanied by a decrease in beneficial bacteria, including those from the Verrucomicrobia phylum [163]. This results in compromised integrity of the intestinal epithelium barrier, fostering increased gut permeability and subsequent systemic inflammation, thereby escalating the risk of developing age-related diseases and premature death [160].

The research indicates that the host’s long-term life history, including dietary habits, can have a substantial effect on the microbiome at later life stages [164]. In a study involving elderly Japanese subjects, researchers examined the correlation between fecal quercetin metabolism, intestinal microbiota composition, and dietary intake. The results indicated that making changes to one’s diet can bring about alterations in the gut microbiome and, in turn, affect polyphenol metabolism, thereby potentially benefiting the health of the elderly [165]. Studies have uncovered compelling evidence suggesting that quercetin, a flavonoid ubiquitously present in propolis, has a pronounced influence on the gut milieu, with consequential effects on the regulation of intestinal microbiota [166]. In mice subjected to a high-fat diet, quercetin restored gut microbiota balance and the associated endotoxemia triggered by Toll-like receptor 4 (TLR-4)-NF-κB signaling pathway activation [167]. Additionally, this propolis constituent suppressed inflammasome response and endoplasmic reticulum stress pathway activation, leading to the reversal of gut–liver axis disturbances [167]. Similar outcomes were observed in a study where propolis supplementation was found to have a stabilizing effect on the intestinal microbiota profile, suppress the TLR4 pathway and proinflammatory mediators in muscle, and decrease blood levels of lipopolysaccharide (LPS) induced by a high-fat diet in rodents [168]. Toll-like receptor 4 (TLR4) signaling is a critical step in the inflammatory cascade induced by LPSs. Immune cells recognize LPSs through TLR4, which undergoes a conformational change that triggers the recruitment of intracellular adaptor proteins, such as MyD88 (myeloid differentiation primary response 88), initiating downstream signaling by involving other proteins. Eventually, this signaling cascade activates the transcription factor NF-κB, which results in the production and release of proinflammatory chemokines and cytokines, like tumor necrosis factor-alpha (TNF-α) and interleukins (IL-1, IL-6, etc.).

The interplay between gut microbiota and the development of obesity, metabolic syndrome, and type 2 diabetes has been the subject of various studies, with a particular focus on changes in the regulation of glucose and insulin sensitivity. SCFAs arise from anaerobic bacterial fermentation in the gastrointestinal tract. They influence the metabolism of lipids and carbohydrates, primarily through the inhibition of glycolysis and by promoting either lipogenesis or gluconeogenesis [169,170]. Maintaining a proper equilibrium between lipogenesis and the oxidative degradation of fatty acids, SCFAs also impact glucose metabolism systemically [170]. SCFAs regulate pathways governing mechanisms, encompassing receptors, synthesis, hormones, and perception, as they target different body organs. Functioning as strong secretagogues for glucagon-like peptide-1 (GLP-1) and peptide YY (PYY), they amplify the sense of satiety through the gut–brain axis. As a result, this may lead to an indirect reduction in appetite and subsequent food consumption [171]. Through their interaction with the free fatty acid receptors FFA2 and FFA3, SCFAs effectively prevent nuclear factor kappa B activation, leading to the suppression of tumor necrosis factor α (TNF-α) and interleukin-6 (IL-6) release [80]. This inhibition is crucial as persistent inflammation can induce insulin resistance in bodily organs, ultimately impairing glucose tolerance. In diabetic rats, propolis treatment led to improvements in the tight junctions and gap junctions in the intestinal epithelium, repairing intestinal mucosal damage [85]. Beyond the elevation of SCFA levels, the study also indicated propolis’ hypoglycemic effects and its positive influence on gut microbial assemblages, restoring the intestinal microbiota microecology of diabetic rats. In line, studies have revealed that propolis administration effectively counteracts dysbiosis, leading to an increase in SCFA production in feces and a decrease in the expression of inflammation-related genes (TNFα and IL-6) in the liver and muscle [80]. These effects provided protection against sarcopenic obesity in Db/Db mice, a model of type II diabetes and obesity. Sarcopenia refers to the natural loss of muscle mass, strength, and muscle function that occurs as a result of aging [172]. Both sarcopenia and cachexia are incapacitating conditions characterized by muscle deficiency, leading to compromised individual function and physical performance.

As the population ages, it is likely that the prevalence of cancer cachexia will increase in the coming years. Experimental data indicate that cancer development causes significant shifts in the intestinal microbiome’s composition, stimulating the release of inflammatory mediators and reactive oxygen species and contributing to the onset of cachexia [173]. Through the modulation of essential factors linked to sarcopenia, such as microbiota dysregulation, inflammation, metabolic disturbances, and oxidative stress, propolis and its active components appear to hold promise in mitigating muscular decline [174]. However, research into their effects on skeletal muscle loss and weakness is still in its early stages.

Osteoarthritis takes precedence as the most prevalent arthritis type in older adults, and it ranks high among the common causes of physical disability in adults. The identification of intestinal microbiota DNA in the synovium of individuals with osteoarthritis piques interest [175]. In light of the connections between gut microbiota imbalances and the development and progression of osteoarthritis, there is ongoing research into the feasibility of using gut microbiota as a therapeutic target for its treatment [175]. In an osteoarthritis rat model, a study highlighted the positive impact of quercetin treatment on mitigating intestinal flora disorder and restoring fecal metabolite abnormalities [166]. Multiple studies have been undertaken to investigate the influence of propolis and its key constituents on gut microbiota in both health and disease settings [53,85,97,98,154,176,177,178,179,180,181,182,183,184,185]. Although early research suggests that propolis may help optimize gut health, more comprehensive studies are needed before clinical recommendations can be made.

## 6. Autophagy

Via the lysosome degradation pathway, autophagy serves as a critical mechanism for maintaining cellular homeostasis through the clearance of intracellular surplus or damaged molecules and organelles [186]. Autophagy enables cells to dispose of and recycle cellular waste, improving cell health and survival during times of stress or nutrient deficiency. It helps the cell to remove dysfunctional or damaged organelles, proteins, and other cellular components, promoting cellular rejuvenation, stress adaptation, and overall health. Autophagy is of utmost importance in multiple physiological processes, comprising development, immunity, and disease prevention (e.g., cancer, neurodegenerative disorders, and infections). Consequently, its abnormal regulation is implicated in aging and a diverse array of human diseases [187]. The growing body of evidence strongly suggests that autophagic activity decreases as individuals age, heightening their susceptibility to age-related diseases [188]. Moreover, autophagy functions as a negative modulator of inflammasomes. There is a bidirectional relationship between inflammatory mediators and autophagy, as each can regulate the other’s activity.

The study of Hsieh et al. points out that Taiwanese green propolis suppresses the NLRP3 inflammasome through autophagy stimulation, leading to a reduction in gouty arthritis inflammation [189]. In mouse macrophages, Brazilian green propolis extract is involved in lowering IL-1β secretion while simultaneously inhibiting caspase-1 activation and NLRP3 inflammasome [190]. The nucleotide-binding oligomerization domain (NOD)-like receptor family of proteins (NLRPs), such as NLRP3 and NLRC4, is recognized for orchestrating the primary innate immune response to cellular stress [191]. In the presence of pathogen-associated molecular patterns (PAMPs) or damage-associated molecular patterns (DAMPs), both NLRP3 and NLRC4 inflammasomes can trigger inflammation and cellular death [192,193]. Once activated, these sensor molecules initiate the formation of the inflammasome complex, which in turn activates caspase-1, an enzyme involved in the production of proinflammatory cytokines such as interleukin-1 beta (IL-1β) and interleukin-18 (IL-18) [194]. Thus, deficiency of autophagy can result in excessive activation of the inflammasome, leading to pathological hyperinflammation. Arias et al. highlighted the beneficial effects of propolis treatment by reverting osteoarthritis-like biological changes induced by IL-1β stimulation in the chondrocytes of rabbits [195]. This bee-derived product reduced the expression of proteins associated with autophagy, such as microtubule-associated protein 1 light chain 3 alpha (LC3) and autophagy-related 5 (ATG5), as well as AKT1, and nitric oxide production. The RAC-alpha serine/threonine-protein kinase (AKT) serves as an upstream controller of autophagy [196]. Autophagy can be modulated by AKT through mammalian target of rapamycin complex 1 (mTORC1), and while AKT is generally known to inhibit autophagy, certain natural components have been observed to induce autophagy alongside an elevation in AKT phosphorylation [195]. Another investigation by Arias and the research team showcased that propolis provides protection to osteoarthritic chondrocytes by modulating microRNAs that govern autophagy proteins, including AKT1, ATG5, and LC3 [197]. Additionally, Arias et al. (2023) also conducted an in vivo study on knee articular cartilage in senescent rats, revealing that propolis treatment ameliorated osteoarthritis severity, improved the structural integrity of cartilage, and increased chondrocyte density [198]. In their work, Wang et al. (2023) detailed the pinocembrin protective effects on cartilaginous endplate chondrocytes [199]. These authors underscored the importance of pinocembrin’s action, specifically through Nrf-2-mediated activation of mitochondrial autophagy and inhibition of ferroptosis in preventing ROS-induced degeneration and calcification of cartilaginous endplate chondrocytes in a model of intervertebral disc degeneration. The nuclear factor erythroid 2-related factor 2 (Nrf2) governs the transcription of numerous genes related to inflammation, redox balance, detoxification, and metabolism [200]. Through its regulatory function, Nrf2 enhances the expression of several detoxifying antioxidant molecules, such as heme-oxygenase-1 (HO-1), NADPH, glutathione S-transferase, and quinone oxidoreductase.

Oxidized low-density lipoprotein (Ox-LDL) plays a critical role in the development of atherosclerosis, an age-related condition, where advancing age acts as an independent risk factor for its progression. Brazilian green propolis exhibits the capability to suppress apoptosis and autophagy triggered by Ox-LDL damage in human umbilical vein endothelial cells (HUVECs) through the PI3K/Akt/mTOR-mediated Nrf2/HO-1 pathway [201]. In another experiment involving HUVECs subjected to lipopolysaccharide, Chinese propolis showed positive anti-inflammatory effects by inhibiting autophagy and the MAPK/NF-κB signaling pathway [202]. The well-established understanding is that multiple molecules, including proinflammatory mediators, redox-sensitive protein kinases, and phosphatases, have the ability to trigger NF-κB signaling and promote ROS generation. As a result, elevated ROS levels serve as a feedback mechanism to further activate NF-κB signaling. Each of these factors can notably trigger the activation of the inflammasome. Additionally, diminished autophagic activity may cause a loss of regulatory control over the NF-κB complex, resulting in its degradation through selective autophagy [203]. Aging-related impairment in autophagy’s clearance function fosters a permissive milieu for the direct or indirect activation of NF-κB signaling, giving rise to an age-associated proinflammatory phenotype that relies on inflammasome activation.

Over the past years, widespread documentation has highlighted the anti-inflammatory and antitumor activities of propolis and its active ingredients [28,204]. Autophagy’s crucial involvement in the favorable outcomes of propolis, particularly targeting the proliferation of cancerous cells within an inflammatory milieu, has been brought to light. Research has provided evidence that propolis, along with its key component caffeic acid phenethyl ester (CAPE), effectively suppresses breast cancer cell proliferation by modulating the TLR4 signaling pathway, leading to the activation of apoptosis and autophagy mechanisms [205]. Propolis and CAPE have the ability to hinder the TLR4 signaling pathway by inhibiting key molecules such as MyD88, TLR4, IRAK4, and TRIF, affecting both the MyD88-dependent and MyD88-independent signaling pathways. The consequential reduction in NF-κB activation and subsequent cytokine release mediated through the TLR4/NF-κB signaling pathway triggers autophagy, contributing to the alleviation of inflammation. Transforming growth factor-β1 (TGF-β1) is known to prompt autophagy in many different cellular systems [206]. TGF-β is renowned for its role in activating the canonical Smad signaling pathway. In line with this reasoning, a study unveiled that through the inhibition of the TGF-β1/Smad3 pathway and the concurrent activation of autophagy, CAPE plays a crucial role in attenuating liver fibrosis [207].

## 7. Mitochondria

Mitochondria are among the early cellular structures affected when cells face detrimental external factors or bioactive agents. Subsequently, they might incompletely reduce molecular oxygen, giving rise to superoxide radicals and becoming the primary source of reactive oxygen species within the cell. Studies have highlighted the importance of propolis extracts as essential scavengers of intramitochondrial ROS, effectively reducing superoxide levels in a concentration-dependent manner [208]. Research yields credible data supporting the implication of oxidants originating from mitochondria and mitochondrial oxidative injury in amyotrophic lateral sclerosis-associated mutant Cu/Zn superoxide dismutase (SOD1) [209,210]. In alignment with this reasoning, evidence has been presented showing that both kaempferol and kaempferide, two flavonoids existing in Brazilian green propolis, possess the capability to restrain mutant SOD1-triggered superoxide production within mitochondria [211]. This underscores the beneficial impact of propolis active ingredients in counteracting neurotoxicity induced by mutant SOD1 in a cellular model.

The β-amyloid peptide-induced toxicity accumulation in the brain contributes to pathogenesis in Alzheimer’s disease. An investigation showed that pinocembrin’s protective effects against Aβ-induced toxicity involve improvements in mitochondrial membrane potential and the suppression of mitochondrial ROS production. It also plays a role in restoring Bcl-2 and cytochrome c levels, while deactivating caspase 3 and caspase 9 [212].

A substantial body of evidence underlines the central role of mitochondrial dysfunction in Parkinson’s disease pathological processes [213,214]. Research indicates that pinocembrin’s aptitude to shield SH-SY5Y cells from Aβ-induced neurotoxicity involves both activating Nrf2/HO-1 pathways and halting mitochondria-dependent apoptosis [215]. The neurotoxin MPP+ operates by inducing the opening of the mitochondrial permeability transition pore, resulting in the release of cytochrome c, with crucial implications for cellular damage and death. Pretreatment of SH-SY5Y neuroblastoma cells with pinocembrin effectively counteracts MPP+-induced mitochondrial malfunctions, encompassing reduced membrane potential, decreased Bcl-2/Bax ratio, and the liberation of cytochrome c [216]. These effects collectively contribute to the maintenance of neuronal viability. In addition to its role in attenuating dopaminergic loss in the MPTP rodent model of Parkinson’s disease, oral administration of CAPE also has the ability to inhibit toxicity induced by the neurotoxin MPP+ in vitro [217]. Furthermore, it directly suppresses the liberation of cytochrome c and apoptosis-inducing factors from mitochondria caused by neurotoxins, underscoring the potent neuroprotective effects of CAPE.

Cerebral ischemia stands as a prominent contributor to global disability and mortality rates. Age stands as a nonmodifiable risk element for cerebral ischemia [218]. The level of adenosine triphosphate (ATP) within cells has been identified as a significant factor in influencing the extent of damage resulting from cerebral ischemia/reperfusion. The process of oxidative phosphorylation in mitochondria contributes to the production of roughly 80–90% of cellular ATP. Through experimentation centered on rat cerebellar neuronal–glial cell cultures exposed to ischemia, researchers found that the application of polyethylene glycol aqueous extract of propolis yielded a noteworthy increase in neuronal viability. This improvement was coupled with a decrease in mitochondrial superoxide levels [219]. Additionally, the extract notably shielded the cultures against hypoxia-induced elevation of inflammatory cytokines like TNF-α, IL-1β, and IL-6 and effectively countered the reduction in both baseline mitochondrial and ATP-coupled respiration rates, resulting in a significant augmentation of the overall mitochondrial respiratory capacity. Another study highlighted that pinocembrin’s protective effects on brain mitochondria are instrumental in enhancing cognitive function in rats exposed to chronic cerebral hypoperfusion. This positive outcome stemmed from the improvement in mitochondrial complex I activity, membrane potential status, alleviation of mitochondrial swelling, and correction of cytochrome oxidase deficiencies [220].

Both heart and liver mitochondria undergo atypical biochemical modifications upon the introduction of antineoplastic agents doxorubicin or vinblastine [221]. These changes include a significant increase in the production of malondialdehyde and anion superoxide, as well as the activation of swelling processes and a worsening of respiratory control. Nevertheless, providing rats with propolis extract prior to antineoplastic agent injections shielded the heart and liver tissues from mitochondrial stress [221]. Studies have suggested that apoptosis stands out as the primary process by which propolis demonstrates its antitumor effects [152,222,223,224]. In the course of apoptosis, the mitochondrial membrane potential is compromised by the establishment of permeability transition pores. These pores arise from the influence of distinct stimuli, initiating intracellular signaling cascades that prompt the diminishment of mitochondrial membrane potential. Propolis has the ability to trigger apoptosis by influencing the Bcl-2/Bax-mediated modulation of mitochondrial membrane potential [225]. The effects of Brazilian green propolis on human lung cancer A549 cells were investigated, revealing its ability to induce apoptosis through a mechanism involving the downregulation of the antiapoptotic gene Bcl-XL and the upregulation of the pro-apoptotic genes Bax and Noxa [226]. The net effect of these changes is the disruption of mitochondrial membrane potential. Research revealed that employing a combination of propolis, thermal cycling-hyperthermia, and low-intensity ultrasound can restrain the growth of pancreatic cancer cell line PANC-1 via ROS-modulated mitochondrial apoptosis [227]. The findings also underscore that mitochondrial dysfunction is regulated through the MAPK pathway, culminating in apoptosis via enhanced poly (ADP-ribose) polymerase (PARP) cleavage. Docetaxel is used extensively in advanced prostate cancer chemotherapy, but a substantial number of patients develop resistance to this treatment. In this context, a study has described the positive effects resulting from the combined treatment of docetaxel with CAPE, leading to apoptosis and metabolism interference in docetaxel-resistant prostate cancer cells [228]. This approach led to a decrease in protein levels of Bcl-2, AKT2, c-Myc, apoptosis and caspase activation inhibitor (AVEN), and pyruvate kinase M2 (PKM2), alongside an increase in protein concentration of Bax, caspase 3, cytochrome c, glucose-6-phosphate dehydrogenase (G6PD), and acylglycerol kinase (AGK).

A research effort involving isolated rat liver mitochondria unveiled that the glycolic extract sourced from Baccharis dracunculifolia, the primary botanical origin utilized by honeybees for crafting Brazilian green propolis, displayed noteworthy capabilities in scavenging 1,1-diphenyl-2-picrylhydrazyl radicals and superoxide anions, along with the ability to chelate Fe^2+^ ions [229]. The treatment also resulted in a decline of basal H_2_O_2_ generation and suppressed the mitochondrial production of ROS triggered by Fe^2+^ or t-BuOOH, one of the most active lipid hydroperoxides in oxidation processes. Additionally, it effectively prevented lipid oxidation within mitochondrial membranes, preserved protein thiol groups, and prevented GSH oxidation. These outcomes underscore the robust antioxidant prowess of this extract, safeguarding liver mitochondria from oxidative harm, and potentially playing a role in the antioxidant and hepatoprotective properties associated with green propolis.

## 8. Nutrient-Sensing Pathways

Cell viability relies on the continuous regulation of energy demands in accordance with nutrient availability. Through the recognition and responsive modulation of fuel substrates such as carbohydrates, lipids, amino acids, and vitamins, the cell can effectively adapt to varying nutrient signals [230]. Nutrient-sensing pathways have evolved to manage cellular energy levels, maintain metabolic stability, and oversee a variety of biological functions. These pathways undergo a loss of regulation and a reduction in their overall effectiveness as individuals age [231]. Nutrient-sensing signaling pathways establish a connection between dietary choices to the aging process. In this regard, calorie restriction has emerged as the most effective approach for promoting a longer, healthier lifespan by regulating these signaling networks across various model organisms [232]. In fact, current findings from longevity research indicate a smaller genetic contribution and a much greater influence of environmental factors than previously assumed [233].

The signaling mechanisms that participate in the detection and interpretation of nutrient and energy levels, encompassing pathways such as insulin and insulin-like growth factor (IGF-1) signaling, as well as those involving mammalian target of rapamycin (mTOR), adenosine monophosphate-activated protein kinase (AMPK), and sirtuins, can provide the molecular groundwork for understanding how lifestyle factors are linked to the process of aging [234].

Insulin/IGF-1 signaling (IIS) nutrient sensing emerged as the first pathway proven to control aging and age-related diseases in organisms [235]. The IIS pathway is essential in managing glucose uptake and glycogen synthesis. It initiates the phosphoinositide 3-kinase (PI3K)/Akt pathway. This intracellular signaling route modulates proliferation, survival, cell growth, and angiogenesis. IIS exhibits regulatory control over FOXO (forkhead box O) transcription factors. When activated, these factors enhance cellular stress resistance, playing a role in the broader context of aging and longevity. Inhibiting FOXO activity via Akt-mediated phosphorylation is a mechanism through which IIS influences processes such as apoptosis and oxidative stress response [236]. The IIS and the PI3K/AKT/mTOR pathways are intricately linked, and their crosstalk is crucial for regulating processes such as protein synthesis, cell growth, and metabolism and maintaining cellular homeostasis [237].

The mTOR plays a crucial role in regulating aging and age-related diseases through its involvement in two separate complexes, known as mTOR complex 1 (mTORC1) and mTOR complex 2 (mTORC2) [238]. Functioning as a controller of protein synthesis, mTORC1 operates by phosphorylating its primary targets, 4E-BP (eukaryotic initiation factor 4E binding proteins) and S6K1 (p70 S6 kinase 1) [239]. Under calorie-restricted conditions, mTOR is downregulated, facilitating an augmentation of catabolic activity. In contrast, in the presence of abundant nutrients, mTORC1 becomes active, stimulating anabolic pathways, including protein, nucleotide, and lipid biosynthesis, while simultaneously hindering processes that govern protein and organelle turnover, such as autophagy and lysosome biogenesis [240]. An excess of nutritional amino acids is tied to aging and diseases. Studies have pointed out that the suppression of mTORC1 by rapamycin can mimic the outcomes of dietary restriction, including the reversal of aging-related phenotype and the promotion of longevity [241,242].

Functioning as an energy sensor, AMPK adjusts cellular metabolism in alignment with fluctuations in energy availability. Through the inhibition of anabolic pathways and the promotion of catabolic processes, AMPK assists cells in coping with energy stress [243]. AMPK also has the ability to suppress proinflammatory signaling pathways, including NF-κB. Moreover, AMPK interacts with sirtuins, particularly SIRT1, establishing a regulatory network that influences how cells respond to stress and energy levels [244]. As NAD(+)-dependent histone deacetylases, sirtuins exhibit a pronounced sensitivity to cellular energy status. Their influence extends to the modulation of cellular metabolism, involving energy production, DNA repair, oxidative stress, glucose homeostasis, and lipid metabolism [245,246]. The extensively researched SIRT1 engages with transcription factors including p53, FOXO, and PGC-1α, which play roles in cellular stress response, apoptosis, and mitochondrial biogenesis. The nutrient-sensing pathways (IIS, mTOR, AMPK, sirtuins) collectively act as key nodes that significantly influence aging and age-related diseases.

Evidence suggests that propolis and its bioactive constituents can influence nutrient-sensing pathways, prompting an examination of their potential therapeutic benefits across different health domains and in addressing age-associated diseases. A study showed that chrysin’s beneficial effects on diabetic nephropathy involve the modulation of lipid metabolism through the activation of AMPK in HFD/STZ-induced diabetic mice [247]. In a parallel manner, a 4-week regimen of chrysin treatment in rats successfully reversed glucose elevation, inflammation, and the decline in serum insulin levels associated with aging, indicating that chrysin helps preserve islet function [248]. The research conducted by Oriquat et al. revealed that chrysin exhibited notable effects in mitigating weight gain and rectifying hyperglycemia, insulin resistance, and dyslipidemia while simultaneously improving adipocytokine levels in rats with metabolic disturbances induced by a high-fat diet [249]. Moreover, the authors provided evidence that chrysin regulates hepatic signaling by activating the AMPK pathway and inhibiting the mTOR and lipogenic pathways, resulting in anti-obesity and antisteatotic effects [249]. The prevalence of nonalcoholic fatty liver disease (NAFLD) is 39% among individuals aged 40 to 50 and exceeds 40% in those aged 70 and above [250]. The pathological manifestations of NAFLD span from simple steatosis to the more intricate necroinflammatory fibrosing disorder recognized as steatohepatitis. Research findings indicate that flavonoids found in propolis possess the ability to alleviate steatohepatitis in HepG2 cells [251]. The hepatoprotective effects of flavonoids entail boosting AMPK activation, mTOR-NF-κBp65 interaction, and PTEN levels. In addition, the results pointed out that naringenin, specifically, developed a stable complex with AMPK, indicating its potential as a viable candidate for NAFLD treatment [251].

In the research conducted by Cardinault and their team, it was suggested that the polyphenols in propolis prompted the transactivation of nuclear factor (erythroid-derived 2)-like 2 (Nrf2), resulting in the amelioration of glucose homeostasis, elevated production of antioxidant molecules, and protection against oxidative stress in diabesity in high-fat-fed mice [252,253]. Another study demonstrated that propolis lowered fat accumulation in the adipose tissues of rats on a high-fat diet by suppressing peroxisome proliferator-activated receptor γ (PPARγ), which plays a role in regulating genes associated with adipocyte differentiation [254]. A research study highlighted the favorable outcomes of Iranian propolis supplementation for individuals with type 2 diabetes mellitus, showing declines in postprandial blood glucose, serum insulin, insulin resistance, and inflammatory cytokines while also indicating elevations in HDL levels [102]. In a study involving nondiabetic volunteers with obesity and insulin resistance, the supplementation of standardized poplar propolis extract powder for a period of three months positively affected insulin homeostasis through its impact on both insulin resistance and secretion [255]. According to this study, propolis may act preventively on the physiopathological mechanisms of type 2 diabetes, potentially hindering the disease’s development.

It has been shown that galangin and pinocembrin contained in propolis promise to alleviate insulin resistance in HepG2 cells by modulating the protein kinase-B (Akt)/mTOR signaling pathway. These substances showcase their efficacy by distinctly boosting the phosphorylation levels of human insulin receptor (IR), Akt, and glycogen synthase kinase-3 beta (GSK3β) while notably reducing the phosphorylation of insulin receptor substrate (IRS) [256]. Another study reported that the potential of Brazilian propolis to prevent hyperglycemia lies in its ability to promote the translocation of insulin-sensitive glucose transporter (GLUT) 4 and enhance glucose uptake [257]. Additionally, it activates the phosphorylation of PI3K and AMPK in skeletal muscle.

Extensive research has delved into chrysin’s capacity to restore insulin sensitivity and its effects on diabetes [248]. As an illustration, chrysin’s positive impact on glycolipid metabolism has been documented by its capacity to activate AMPK, thereby regulating the PI3K/AKT signaling pathway. This effect was observed in studies using high-fat diet/streptozotocin (STZ)-induced C57BL/6J mice [258].

The expression of LOX-1, the receptor for oxidized low-density lipoprotein (ox-LDL), is heightened in diseases such as atherosclerosis, hypertension, diabetes, and others, facilitating the production of ROS. Chinese popular propolis, as evidenced by Chang et al.’s study, was found to alleviate injury in endothelial cells induced by oxidized low-density lipoprotein. This protective mechanism encompassed the activation of the PI3K/Akt/mTOR signaling pathway, inhibition of LOX-1/p38 MAPK, suppression of ROS production, and preservation of mitochondrial membrane potential to hinder apoptosis and autophagy [259]. These findings present a novel perspective for understanding the mechanisms that contribute to the protective actions of propolis against endothelial apoptosis. In a rat model of diabetes-induced atherosclerosis initiated by HFD/STZ administration, quercetin exhibits the potential to improve atherosclerotic pathophysiology by stimulating AMP activation. This crucial regulatory protein in glycolipid metabolism functions by elevating downstream SIRT1 protein levels and diminishing NF-κB, thereby mitigating inflammatory and oxidative stress responses [260]. Studies have provided evidence suggesting quercetin as a potential therapeutic agent for mitigating the damage induced by myocardial ischemia through significant upregulation of SIRT1, peroxisome proliferator-activated receptor-γ coactivator-1α (PGC-1α), and Bcl-2 proteins, accompanied by a reduction in Bax protein expression [261].

Research has also underscored quercetin’s capacity to mitigate mitochondrial dysfunction and promote biogenesis in osteoarthritis-afflicted rats by increasing the expression levels of SIRT1, PGC-1α, NRF1, NFR2, TFAM, and phospho-AMPKα, suggesting its action through the AMPK/SIRT1 pathway [262,263]. Taken together, studies have suggested quercetin’s capacity to serve as a promising therapeutic option to tackle age-related diseases through its engagement with SIRT1 pathways [264,265,266]. Current research findings have indicated that quercetin exerts protective roles in age-associated diseases by activating SIRT1, resulting in the coordination of a broad spectrum of cellular processes linked to the aging phenomenon. These processes encompass oxidative stress through SIRT1/Keap1/Nrf2/HO-1 along with PI3K/Akt/GSK-3β pathways, inflammatory reaction via SIRT1/NF-κB signaling, mitochondrial status via SIRT1/PGC1α/eIF2α/ATF4/CHOP, and autophagy through the SIRT1/FOXO pathways [266]. Through their research on prostate cancer cells, Tseng et al. discovered that CAPE treatment effectively suppressed PI3K-Akt signaling, a pathway often hyperactive in cancers and fostering tumor progression. These results underscore CAPE’s potential as an inhibitor targeting the PI3K/Akt pathway, presenting novel avenues for cancer treatment research [267]. Havermann et al. showed that CAPE enhances resistance to thermal stress and extends *Caenorhabditis elegans*’s lifespan through the regulation of the DAF-16 signaling pathway [268]. The authors detailed that CAPE induces the nuclear translocation of DAF-16, a critical step for the extension of the nematode’s lifespan. In *Caenorhabditis elegans*, DAF-16 is the unique ortholog representing the FOXO proteins family. It stands as the key downstream target and primary effector in the DAF-2/insulin-like signaling pathway [269]. In this line of research, a significant lifespan-extending effect in *Caenorhabditis elegans* has been observed through the administration of Okinawa propolis, a resinous mixture produced by honeybees [8]. Taira et al. found that Okinawa propolis, in addition to promoting nematode’s longevity, demonstrated an antimelanogenic effect in melanoma cells and anticancer properties against lung cancer cells. All these effects of propolis were attributed to the inhibition of the oncogenic/aging/melanogenic p21-activated kinase (PAK1) [8].

## 9. Intercellular Communication

Intercellular communication is an integrative hallmark of aging, involving the exchange of signals between cells through various mechanisms, including direct cell-to-cell contact, secretion of signaling molecules (such as hormones, growth factors, cytokines, and neurotransmitters), and extracellular vesicles (such as exosomes) [270]. During aging processes, cells secrete a range of proinflammatory cytokines, growth factors, and matrix remodeling enzymes as part of the senescence-associated secretory phenotype (SASP) [270]. Studies have established that these factors can prompt neighboring cells to enter a senescent state, potentially accelerating aging and playing a role in the onset of various age-associated diseases [271].

As individuals grow older, the signaling network of chemical messages throughout the body gives rise to a continual, low-level systemic inflammation, coupled with the ongoing activation of the innate immune system. This phenomenon, termed ‘inflammaging’, represents a smoldering proinflammatory phenotype that hastens the aging process and is linked to the development of numerous age-related diseases [23,272]. Several distinct factors converge to induce inflammaging, and one of them is the SASP, which emerges as a direct outcome of another signature of aging—cellular senescence.

Immunosenescence is a term that summarizes the amplification of the aging phenotype, characterized by changes in immune system function. These shifts involve disturbances in T cell populations, a decline in the ability to respond to antigens, a reduced capacity to clear defective host cells, and the lingering presence of low-grade inflammation [273]. The recognition of disturbed intercellular communication as a contributor to aging is growing. In particular, impaired neurohormonal signaling and disrupted inflammatory pathways, in conjunction with an ineffective immune system, have been implicated in this context [234]. Researchers are pursuing approaches to adjust dysregulated intercellular communication pathways in an experimental setting. For instance, administration of anti-inflammatory and anti-oxidant agents, dietary restriction, and the modification of the gut environment seem important in this vein.

In this context, Gao et al. showed that Brazilian green propolis supplementation counteracts age-associated immune changes in aged mice [274]. This is evidenced by increased serum IgG levels, improved specific antibody response, and enhanced phagocytic ability.

Zhu et al. (2018) conducted a study revealing that Mini-Mental State Examination (MMSE) scores of elderly individuals residing in high-altitude areas declined, suggesting mild cognitive impairment over a 24-month period [275]. This decline was associated with an elevation in serum inflammation markers. Interestingly, individuals with prolonged consumption of Brazilian green propolis exhibited a significant decrease in systemic inflammation and demonstrated improved MMSE scores [275]. Other research indicated that Brazilian green propolis treatment in an animal model of Alzheimer’s disease demonstrated efficacy in reducing the elevation of IL-6 inflammatory cytokine levels in plasma, concurrently dampening the immune response in glial cells [49].

The research conducted by Ali et al. documented the protective effects of a standardized pomegranate and propolis blend in Parkinsonian rats. The study indicated that, apart from alleviating motor and cognitive deficits, the treatment increased striatal concentrations of dopamine, norepinephrine, and serotonin. It also suppressed lipid peroxidation, enhanced total antioxidant capacity, restored SOD, and concomitantly reduced caspase-3 as well as inflammatory markers IL-1β, TNF-α, and iNOs [276].

The administration of a drug combination consisting of quercetin and dasatinib, a tyrosine kinase inhibitor, effectively counteracts age-related increases in senescence and inflammation within the adipose tissue of old mice [277]. This treatment not only reduces the age-related elevation of senescence-associated β-galactosidase but also diminishes the expression of aging-related genes such as p16 and p21. Moreover, the cocktail attenuates the upregulation of proinflammatory SASP genes, including mcp1, tnf-α, il-1α, il-1β, il-6, cxcl2, and cxcl10 while concurrently suppressing the infiltration of T cells and macrophages in perigonadal white adipose tissue [277]. Research findings indicate that quercetin enhances cell-to-cell communication by promoting the expression of gap junction proteins, which play a crucial role in facilitating communication between adjacent cells [278]. Through the upregulation of connexin43, quercetin effectively inhibits the proliferation of metastatic human breast tumor cell lines [278].

## 10. Proteostasis

Research conducted in recent decades has brought to light the cell’s remarkable capacity to uphold proteostasis in the face of diverse and demanding circumstances. The significance of this capability becomes especially apparent as we gain a deeper comprehension of the fact that a disruption in this process is a root cause of numerous age-related conditions. In mammalian cells, protein homeostasis, also referred to as proteostasis, is a complex process responsible for maintaining the proteome’s stability through the management of protein synthesis, folding, trafficking, post-translational modifications, and degradation. An intricate and adaptable proteostasis network oversees these functions with the involvement of diverse molecular chaperones, along with the ubiquitin–proteasome and lysosomal–autophagy systems. Their interplay preserves the integrity and operational efficiency of the cellular proteome while reducing the likelihood of misfolding or aggregation, which serve as prominent indicators of age-related proteinopathies [279,280]. Scientific investigations have suggested that low doses of CAPE can produce favorable effects in addressing disorders marked by protein aggregation through the activation of hypoxia-inducible factor-1α (HIF-1α) and the suppression of stress-induced protein aggregation [281]. The endoplasmic reticulum is pivotal for maintaining protein quality through the initiation of the unfolded protein response (UPR) [282]. In eukaryotic systems, glucose-regulating protein 78 (GRP78), a chaperone protein within the heat shock protein family (HSPA5), assumes the central role in regulating the UPR that takes place within the endoplasmic reticulum’s lumen [283]. Hirata et al.’s research (2021) uncovered the ability of artepillin C to suppress both neuronal cell death related to ER stress and protein aggregation. Its effectiveness was shown through the inhibition of tunicamycin-induced protein aggregation in the hippocampal cell line HT22 and the spontaneous protein aggregation of mutant canine superoxide dismutase 1, akin to human amyotrophic lateral sclerosis, in Neuro2a cells [284]. The study indicated that artepillin C has the ability to inhibit the protein kinase R-like endoplasmic reticulum kinase (PERK) and inositol-requiring enzyme 1 (IRE1) branches within the ER stress pathway [284]. Artepillin C also had a significant impact on reducing the levels of growth arrest and DNA damage-inducible gene 153 (GADD153) and spliced X-box binding protein 1 (XBP1) induced by tunicamycin. Hirata et al.’s study implied that artepillin C displays chemical chaperone-like properties. The PERK, IRE1, and activating transcription factor 6 (ATF6), positioned in the ER membrane, serve as the protein sensor activators directing UPR regulation [285]. Under physiological conditions, the binding immunoglobulin protein (BiP), also known as GRP78, represses the activity of these sensors, binding them. The accumulation of unfolded/misfolded proteins in the ER results in the release of BiP from stress sensors. Once released, each protein initiates a different regulatory mechanism that increases protein-folding capacity and reduces protein load on the ER. Activation of PERK results in the inhibition of the eukaryotic initiation factor 2α (eIF2), leading to a global inhibition of protein synthesis [286]. PERK enables the translation of GADD153, inducing cell death in response to ER stress. In turn, the cytosolic portion of IRE1 contains a kinase and an endoribonuclease domain, both of which are required for the unconventional splicing of X-box binding protein 1 (XBP1) messenger RNA (mRNA). The spliced XBP1 mRNA encodes a functional transcription factor that translocates to the nucleus, regulating the expression of genes involved in protein folding, folding quality control, ER-associated degradation (ERAD), and lipid biosynthesis. Research has revealed that the ethanol extract of propolis is proficient in preventing the activation of the endoplasmic reticulum stress signaling pathway triggered by oxidized low-density lipoprotein or tunicamycin. This inhibition encompasses the phosphorylation of double-stranded RNA-activated PERK and eIF2α, together with the elevation of GRP78 and CHOP, a pro-apoptotic molecule [287]. In their 2017 study, Tomiyama et al. showed that pretreating human SH-SY5Y neuroblastoma cells with CAPE can trigger endoplasmic reticulum (ER) stress and subsequent autophagy, playing a role in cell survival following cytotoxic injury caused by the neurotoxin 6-hydroxydopamine (6-OHDA) [288]. The authors posited that autophagy represents a mechanism implicated in the protective effects of ER preconditioning. Emerging findings indicate that the initiation of the UPR in Parkinson’s disease critically relies on the activation of the chaperone GRP78/binding immunoglobulin protein (BIP). This highlights that neuroprotective substances that can modulate the expression of GRP78/BiP have the potential to regulate endoplasmic reticulum stress in experimental models of this neurodegenerative condition [289]. Current research has presented findings on the ability of polyphenol-based nanosheets of propolis to regulate protein aggregates implicated in neurodegenerative conditions. A study has provided evidence of the polyphenolic fraction of propolis nanosheets binding to α-synuclein fibrils while also showcasing substantial dose-dependent inhibition of α-synuclein aggregation by these nanosheets [290].

Chrysin exerts its pro-death effects on prostate cancer cells by inducing mitochondrial-mediated apoptosis and endoplasmic reticulum stress, the latter driven by the activation of UPR proteins like GRP78, PERK, and eIF2α [291]. The disruption of mortalin-p53 complexes is one of the mechanisms underlying CAPE’s anticancer and antimetastasis properties [292]. Mortalin is an hsp70 chaperone abundantly found on the surface of cancer cells. To harness this knowledge, scientists employed a specialized cell-internalizing antimortalin antibody to engineer mortalin-targeting CAPE nanoparticles. These innovative nanoparticles demonstrated heightened selective anticancer efficacy in both in vitro and in vivo evaluations, indicating their potential as a potent nanomedicine for combating cancer [293]. Akin to CAPE, artepillin C also induces the activation of p53’s tumor suppressor by disrupting its binding with mortalin [294]. Evidence supports CAPE’s capacity to hinder E6-associated protein (E6AP), thereby disrupting the interaction between p53 and the E3 ubiquitin ligase E6AP. As a result, the ubiquitination of p53 is prevented, underscoring CAPE’s anticervical cancer properties [295].

## 11. Epigenetics

Epigenetic regulation is the process of governing gene expression while keeping the actual genetic code unchanged. Epigenetic markers function as genetic regulators, dictating which segments of DNA are accessible for transcription [296]. The primary epigenetic changes involve DNA methylation, modification of histone proteins, and RNA-mediated processes that impact gene expression. These changes are heritable and often reversible [297]. They are significant contributors to variations in gene expression patterns among different tissues and can also exert influence over gene expression across different age groups. Epigenetic changes are susceptible to environmental influences, functioning as a liaison between gene expression and the environment [298]. Hence, environmental factors like diet or medications can govern epigenetic adjustments, providing a means for the environment to dictate an organism’s genetic constitution. As an organism progresses through its life, the scale, kind, and distribution of epigenetic adjustments shift, causing variations in gene expression that may have adverse effects on the organism. Such epigenetic changes have the potential to disrupt cellular function and foster diseases [299,300]. An increasing number of epigenetic modifications have been documented in the control of critical genes relevant to aging and its associated diseases, such as cancer. In this context, substances that possess epigenetic modulation capabilities, particularly inhibitors of histone deacetylases and DNA methyltransferases, have garnered expanded interest in the domains of aging and cancer research.

It is widely recognized that, apart from genetic mutations, epigenetic changes also accrue as people age, potentially contributing to the process of carcinogenesis. As an example, telomerase and p16INK4a (p16, cyclin-dependent kinase inhibitor 2A) are representative epigenetic targets that feature in both aging and cancer scenarios [301]. In this context, the focus is on substances able to change epigenetic processes. Naturally derived substances such as propolis and its components have the potential to influence or suppress epigenetic modifiers. A case in point is quercetin, a dietary polyphenol acknowledged as an agent capable of modulating epigenetic processes [301]. It is established that T24 human bladder carcinoma cells display hypermethylation of genes associated with cell cycle regulation, particularly p16INK4a and RASSF1A. Quercetin-induced demethylation of these genes restores their transcriptional function, suggesting their role in impeding cell growth and promoting apoptosis in T24 cells after exposure to quercetin [123].

Tumorigenesis is marked by the critical involvement of activated NF-κBp65 (RelA) and aberrant histone deacetylase (HDAC) activity [302]. The use of HDACs is on the rise as a developing strategy to impede the NF-κB pathway, with their modulation of NF-κB activity centered on the RelA/p65 subunit [303,304]. In the investigation conducted by Zheng et al. (2014) using human esophageal cancer cell line EC-9706, it was found that quercetin treatment resulted in the inhibition of RelA/p65 activity, downregulation of HDAC1, RelA, and cyclin D1, and upregulation of caspase-3 and p16INK4α [305]. The literature indicates that the regulation of COX-2 expression upon exposure to proinflammatory mediators involves transcriptional activation of NF-κB, CCAAT/enhancer binding protein (C/EBP), and CREB-2 [306]. In line, studies have also provided insights into how quercetin regulates COX-2, a pivotal mediator involved in inflammation and tumorigenesis. The work of Xiao et al. revealed a marked inhibition of COX-2 mRNA and COX-2 promoter activation in breast cancer cells upon treatment with quercetin [307]. Additionally, they outlined quercetin’s substantial inhibition of key drivers involved in COX-2 transcriptional activation (NF-κB, CREB2, and C/EBPβ), thereby impeding the recruitment of the coactivator p300 histone acetyl transferase to the COX-2 promoter [307]. These findings hint at quercetin’s potential application in the treatment of COX-2-associated diseases.

Studies have shown that quercetin exerts control over the expression of diverse chromatin-modifying factors and reduces the activity of class I/II histone deacetylases, DNA methyltransferases 1, and histone methyltransferases in human cervical cancer (HeLa) cells; the extent of the modulation varies with the dose [308]. In addition, this intervention yielded reduced overall DNA methylation levels, while tested tumor suppressor genes exhibited a significant dose-dependent reduction in promoter methylation, leading to the reinstatement of their expression [308]. The influence of propolis and its bioactive components, with a particular emphasis on quercetin, in shaping microRNA expression presents an engaging avenue for regulating inflammation, differentiation, proliferation, apoptosis, and immune responses [197,309,310,311,312,313].

The phenomenon of postovulatory aging is acknowledged for its detrimental impact on both oocyte quality and the development of ensuing embryos, representing a major impediment to the success of human assisted reproductive technology. Despite this, the options for counteracting the deterioration of aged oocytes are limited in scope. Experimental data have pointed out that quercetin can postpone the postovulatory aging of mice oocytes. The treatment involving this propolis component mitigated the age-related irregularities in the way spindles were organized and mitochondria were distributed, all the while preserving SIRT expression and histone methylation [314]. Nonetheless, our insight into the role of epigenetic mechanisms in these strategies for addressing aging is in its early stages [300].

## 12. Shortcomings and Perspectives

Available data suggest that characteristics of aging serve as primary catalysts behind aging-associated diseases. The significance of aging hallmarks becomes more pronounced when considered as an interlinked network rather than isolated biological processes. Propolis’s complex chemical composition equips it with a spectrum of biological attributes and multifaceted pharmacological effects. Propolis exhibits the capability to simultaneously interact with multiple biological targets, inducing pleiotropic effects. Therefore, grasping the mechanisms of propolis action poses a significant challenge, requiring the identification of primary targets and an understanding of the interconnected networks of diverse signaling pathways. Currently, computational frameworks empower the integration of extensive and diverse sets of biological data. Artificial intelligence algorithms excel in the analysis of integrated data, pinpointing patterns and relationships. Additionally, these algorithms can appraise molecular structures and evaluate binding affinities and pharmacological properties. By modeling the relationships between drug candidates (propolis and its bioactive compounds), targets, and pathways, researchers can gain insights into the multifaceted mechanisms of drug action. Moreover, computational tools are essential to systems biology approaches, which model the behavior of entire biological systems. These models enable the simulation of propolis’s impact on signaling pathways and cellular processes, offering a comprehensive outlook on its mechanism of action in the context of aging and age-related diseases.

Although propolis and its components hold promise as potential interventions that can address these fundamental facets governing both aging and age-related diseases, this research topic is in its infancy, and considerable work is still needed to gain a comprehensive grasp of their mechanisms of operation. Attention is drawn to the imperative of standardizing this bee-derived product while implementing rigorous quality control measures. Certain researchers have already taken steps in this direction, particularly in relation to Brazilian green propolis [315] and propolis of the poplar variety [316,317,318]. It is only when such standardization is achieved that this natural product can truly serve as a viable adjunctive therapy with internationally recognized quality control standards.

While evidence has emerged from preclinical studies, more rigorously designed clinical trials involving humans are essential to determine the efficacy and safety of propolis in addressing age-related diseases and the aging process. For a comprehensive assessment of the mechanisms of action and biological effects of propolis and its constituents, studies should cover different life stages. It is of great significance to note that older adults are frequently overlooked in research endeavors. The assessment of the long-term safety of propolis supplementation, especially in older individuals, is vital to verify the absence of adverse effects linked to its extended use. There is a need to explore potential interactions between propolis and frequently prescribed medications, particularly for individuals dealing with various health conditions. Moreover, efforts should be intensified to encourage multicenter collaborations.

It is of utmost importance to determine the scope of benefits of propolis and its compounds and optimize their therapeutic capabilities. The refinement of delivery strategies and chemical adjustments, resulting in the development of more powerful compounds with superior pharmacodynamic and pharmacokinetic profiles, holds the potential to advance the utilization of these natural bee-derived products as effective interventions for humans. In this scenario, the work by Takahashi et al. showed that the esterization of bioactive compounds from propolis with the water-soluble 1,2,3-triazolyl alcohol resulted in a 30-fold augmentation of artepillin C’s anti-oncogenic P21-activated kinase1 (PAK1) activity, and CAPE exhibited a remarkable 140-fold enhancement [319]. This led to noteworthy improvements in both anticancer activities and cell permeability of these propolis compounds. Moreover, the research of Vasileva et al. uncovered the potential anti-aging effects of the natural deep eutectic extracts (NADES) of propolis in mutant S. cerevisiae yeast strains, which manifest premature aging features [320]. NADES are formed by primary metabolites derived from plants. Their distinctive features, including biodegradability and biocompatibility, position them as alternative options in various concepts and applications that traditionally employ organic solvents. In addition to their nontoxic attributes, NADES have the potential to amplify the biological activities of the extracted compounds [321].

Taken together, the continuous growth of nanomedicine and food nanotechnology, along with the advancements in bioinformatics and artificial intelligence technologies over the last few years, has opened up opportunities for the establishment of new approaches for dietary assessment and the exploration of physiological outcomes [322,323,324,325]. Additionally, there is an expectation that the incorporation of cutting-edge methodological approaches, including multi-omics strategies like transcriptomics, metabolomics, proteomics, genomics, epigenomics, and microbiomics, will propel advancements in this area.
